# Peripheral-central correlation study of acupuncture for chronic tinnitus study protocol for a randomized controlled trial

**DOI:** 10.3389/fmed.2025.1543023

**Published:** 2025-02-13

**Authors:** Da Jiang, Xiao-Han Huang, Ke Fang, Ming-Hui Zhao, Yang Li, Han-Tong Hu, Lian-Qiang Fang, Hong Gao, Jie Zhou

**Affiliations:** Department of Acupuncture and Moxibustion, The Third Affiliated Hospital of Zhejiang Chinese Medical University, Hangzhou, China

**Keywords:** tinnitus, acupuncture, protocol, randomized controlled trial, chronic tinnitus, ABR, fNIRS

## Abstract

**Purpose:**

(1) Exploring the evaluation and correlation of peripheral central auditory function in patients with chronic tinnitus. (2) Evaluation of the cumulative effect of acupuncture on peripheral central auditory function in patients with chronic tinnitus.

**Method:**

Our research is structured as a regulated and randomized trial with assessor blinding. Seventy-two participants who qualify with chronic tinnitus will be allocated in a 1:1 ratio to either the acupuncture group or the sham acupuncture group. Additionally, we will recruit 15 healthy individuals as subjects for data collection to observe the correlation of peripheral-central auditory function under different physiological states.

**Result:**

Clinical result metrics encompass the Tinnitus Handicap Inventory (THI), ABR testing, and fNIRS data collection. Evaluations will be carried out at baseline, after 10 treatment sessions.

**Conclusion:**

This research are anticipated to improve our comprehension of the effectiveness and fundamental processes of acupuncture in addressing persistent tinnitus and deeply explain the mechanism of action of the acupuncture method on chronic tinnitus.

**Clinical trial registration:**

ClinicalTrials.gov, identifier NCT06401993.

## Introduction

Tinnitus is the perception of one or more sounds without the presence of an outside sound source (1). The latest European tinnitus guidelines consider a duration of up to 3 months as the acute phase, 3–6 months as the subacute phase, and over 6 months as the chronic phase (2). The global prevalence of tinnitus ranges from 5.1 to 42.7%, and the incidence rate in mainland China is consistent with the global data, at 4.3–51.33% (3). Chronic tinnitus can impact individuals’ employment, auditory perception, emotional state, and sleep patterns, while severe cases may result in psychiatric disorders and even suicidal tendencies (4). Due to the unclear pathogenesis of tinnitus, current clinical treatments mainly include pharmacotherapy, sound therapy, and habituation therapy, however, there is no conclusive evidence to support the efficacy of these treatment options (2, 5), hence most patients with tinnitus progress to the chronic phase. Therefore, delving into the key mechanisms underlying chronic tinnitus and seeking effective treatment methods has always been a hot topic in auditory neuroscience research.

The occurrence of tinnitus is related to abnormal auditory nerve function. Some studies have reported that changes in the excitation threshold of cochlear hair cells may lead to tinnitus (6), especially when there is a decrease in the number of ribbon synapses on cochlear hair cells. This results in a reduction in the level of neurotransmitter release in the synaptic cleft, a decrease in signals transmitted to the auditory central system, and an abnormal increase in central excitability, leading to tinnitus (7). It is important to note that tinnitus is not solely caused by changes in cochlear activity but also originates from higher-level structures within the auditory nerve pathway. The removal of the cochlea or dorsal cochlear nucleus in guinea pigs after long-term exposure to noise does not alter the hyperpolarized electrical activity of the auditory center, indicating that tinnitus can exist independently without the influence of the periphery (8). Clinically, it has also been reported that long-term tinnitus patients do not experience a disappearance of tinnitus symptoms after surgical transection of the cochlear nerve (9). In addition, recent studies have shown that brain structures outside the auditory nervous system, including the limbic system, parietal lobe, and frontal lobe, are also involved in the maintenance process of tinnitus (10, 11). These areas may mediate the perception of tinnitus through their influence on cognition, emotion, and behavior (12). Therefore, it is currently believed that the periphery (cochlea) is the initial site of tinnitus generation, and the central plasticity changes it triggers are key factors in the occurrence of tinnitus. Hence, tinnitus is also considered a “central reorganization disease” (13).

Auditory brainstem response (ABR) is a bioelectric response of the brainstem evoked by sound stimulation, reflecting the highly synchronized activity of the auditory center from the cochlea to the brainstem. It can sensitively locate damage to the auditory nerve and brainstem conduction pathways (14). ABR consists of seven waveforms, among which waves I, III, and V are the most stable and prominent in the ABR waveforms. In animal models, it has been confirmed that the amplitude of wave I of ABR is related to the number of ribbon synapses in the cochlear hair cells and nerve fibers (15). In clinical trials, it has also been confirmed that the amplitude of wave I of ABR is related to tinnitus symptoms (16). Therefore, ABR can objectively reflect the patency of the peripheral auditory conduction pathway and the normality of brainstem function, and can be used as an objective evaluation index for tinnitus. However, the relationship between ABR and tinnitus still needs further clarification.

Functional Near-Infrared Spectroscopy (fNIRS) is a new non-invasive technique for brain functioning imaging that has emerged in recently. fNIRS can immediately and in real-time ascertain the cognitive cortex’s hemodynamic activity, and infer the neural activity of the brain based on this. In recent years, researchers have begun to use fNIRS to explore the mechanisms related to tinnitus, and preliminary findings have shown that patients with tinnitus have abnormal neural functional connectivity strength between the auditory center and various cerebral areas such as the temporal–parietal and parietal lobes (17). Numerous studies have confirmed that the brain activity obtained using fMRI is highly correlated with the fNIRS signals in the same region, indicating the authenticity and accuracy of the detection results of fNIRS (18, 19). Moreover, compared with fMRI, fNIRS is more sensitive to changes in functional activity of the cerebral cortex, has a higher temporal resolution, and can track changes in brain functional activity at all times and throughout the process. Most importantly, its silent examination characteristic makes it more suitable as an examination method related to hearing.

Acupuncture is a convenient and low-side-effect complementary therapy that has long been used to treat tinnitus, especially in China. Some systematic reviews and meta-analyses generally support the effectiveness and safety of acupuncture in treating tinnitus (20–23). With in-depth research, more and more evidence suggests that acupuncture can significantly improve tinnitus symptoms (24, 25). Studies have also found that acupuncture can improve tinnitus by regulating central neurotransmitters, such as gamma-aminobutyric acid and serotonin and their receptors, thereby inhibiting or activating specific components of the auditory system (26). Recently, literature has reported that acupuncture at corresponding acupoints can enhance the blood oxygen content in the auditory cortex and activate the auditory cortex of tinnitus patients, as observed by fNIRS, preliminarily elucidating its mechanism of action in treating tinnitus (27). However, there is currently a lack of sufficient evidence to support the exact mechanism of acupuncture in treating tinnitus, especially the relationship between the therapeutic effects of acupuncture and the auditory conduction pathway and brain functional activity in tinnitus is still unclear and requires further in-depth research. Moreover, due to tinnitus being a patient-reported subjective symptom, there are no objective evaluation techniques available to objectively analyze the healing benefits of individual remedies for improving tinnitus. In light of these study deficiencies, functional near-infrared spectroscopy (fNIRS) and auditory brainstem response (ABR) may yield significant insights.

Given the significant potential of fNIRS and ABR in tinnitus research, and the constraints of prior studies, we have developed this randomized controlled experiment (RCT) to investigate the pathological mechanisms of chronic tinnitus using fNIRS and ABR. The objectives of this trial are: (1) To compare the auditory brainstem response parameters and extracerebral oxygen levels of the cerebral cortex between chronic tinnitus patients and healthy subjects, and to compare the correlation of peripheral-central auditory function under different physiological states. (2) To compare the intervention effects of acupuncture versus sham acupuncture on chronic tinnitus patients from clinical manifestations, ABR parameters, and extracerebral oxygen levels of the cerebral cortex, exploring the correlation of acupuncture intervention on chronic tinnitus from the peripheral-central auditory function at three levels: clinical, target organ, and central.

## Materials and methods

### Study design

**Figure 1:**
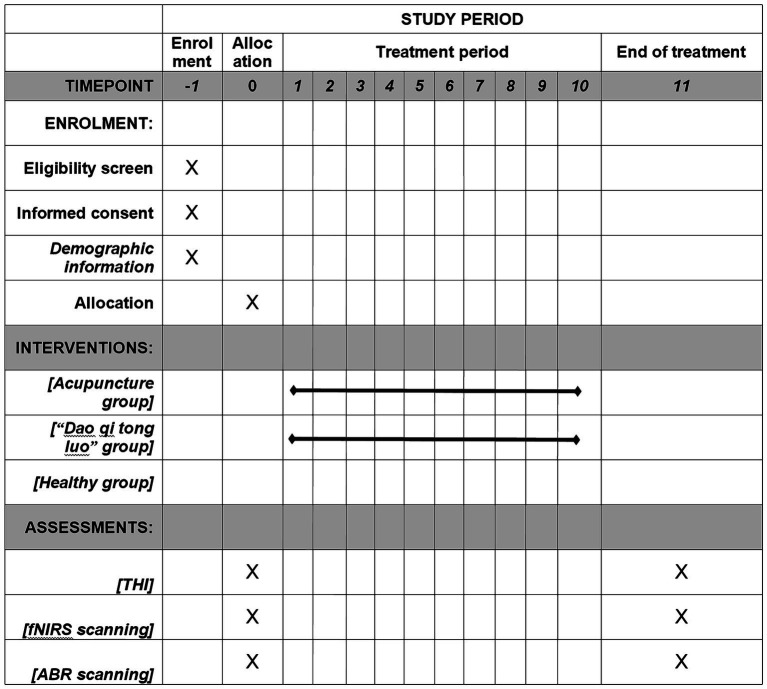
The SPIRIT schedule of enrollment, interventions, and assessment.

**Figure 2:**
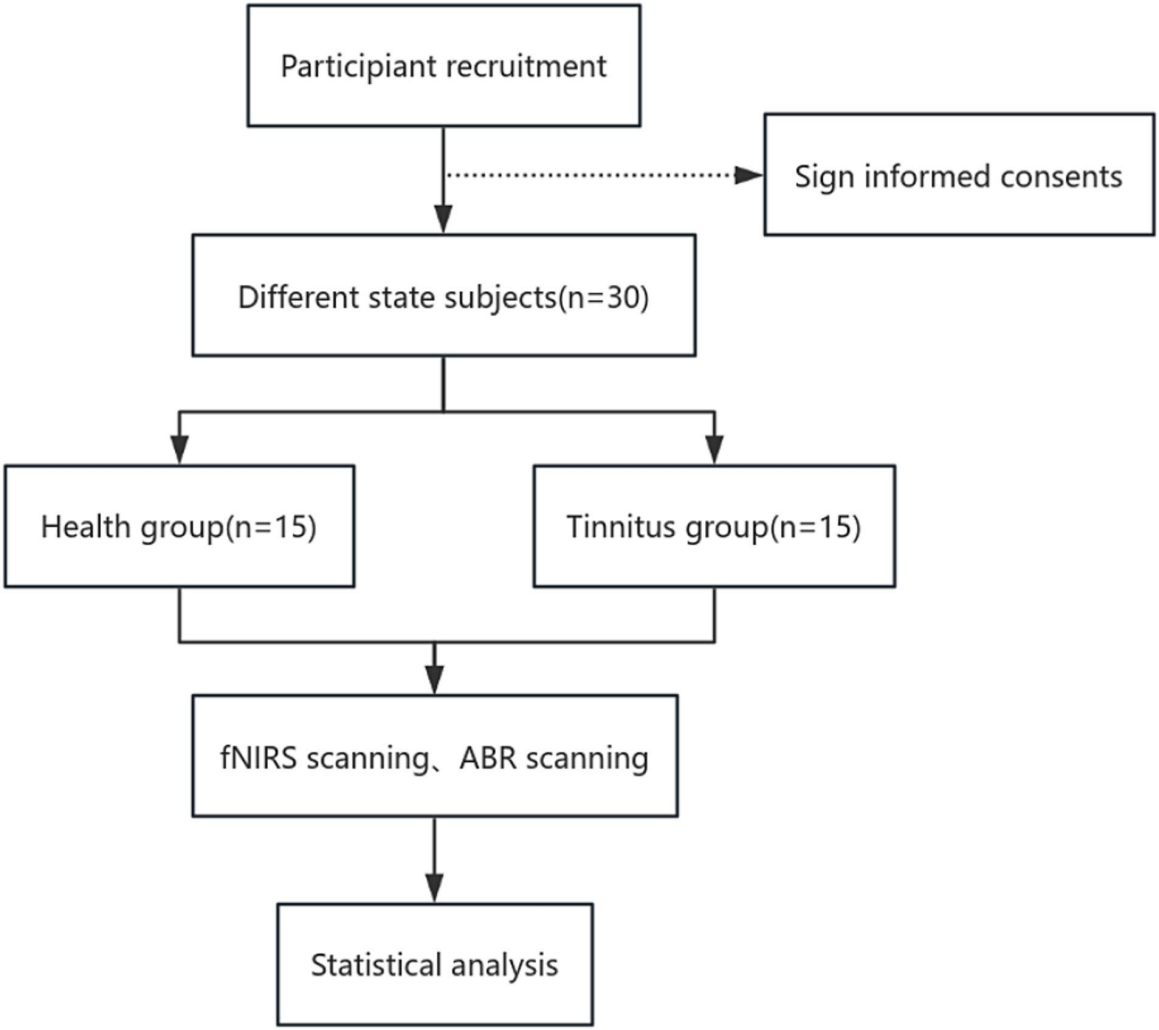
Part one flow chart of the study process.

**Figure 3:**
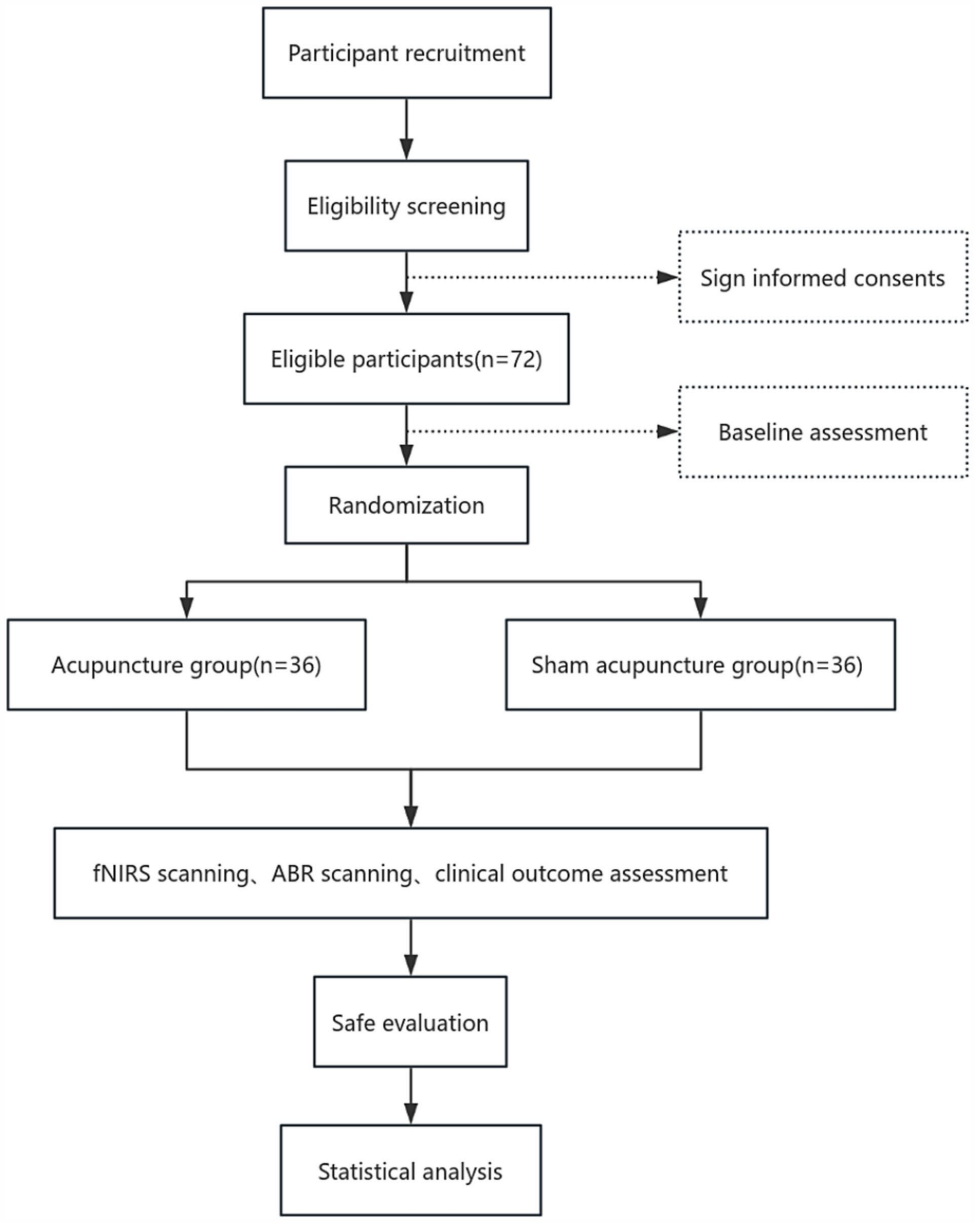
Part two flow chart of the study process.

Our research is structured as a regulated and randomized trial with assessor blinding. Seventy-two participants who qualify with chronic tinnitus will be allocated in a 1:1 ratio to either the acupuncture group or the sham acupuncture group. Additionally, we will recruit 15 healthy individuals as subjects for data collection to compare the auditory brainstem response parameters and extracerebral oxygen levels of the cerebral cortex between chronic tinnitus patients and healthy subjects, and to observe the correlation of peripheral-central auditory function under different physiological states. Our protocol’s reporting complies with the Standard Protocol Items: Recommendations for Interventional Trials (SPIRIT) criteria. The SPIRIT for the study is shown in Figure 1, and the flowchart is shown in Figures 2, 3.

### Participant recruitment

Individuals who satisfy the eligibility requirements for chronic tinnitus will be recruited from the outpatient departments of Otorhinolaryngology and Acupuncture at the Zhejiang Chinese Medical University’s third affiliated hospital. Before signing the informed consent form, each participant will receive a comprehensive explanation of the protocol.

#### Diagnostic criteria

Diagnosing tinnitus follows international criteria, which characterize it as the perception of sound without an external auditory stimulus. Additionally, according to the multidisciplinary European guidelines on tinnitus, tinnitus is referred to as “chronic” when its duration is 6 months or longer (2).

1. Unlike objective tinnitus, subjective tinnitus can only be perceived by the patient and cannot be assessed via auscultation by a physician.
2. Patients perceive ear noises without the assistance of an outside source; these noises may be continuous or sporadic and can exhibit diverse auditory characteristics, including ringing, buzzing, clicking, or roaring.
3. Otoscopic examination reveals no abnormalities in the ears, encompassing the eardrum and canal.
4. Tinnitus affects the patient’s the norm of living to some extent, and the degree of annoyance varies from person to person.

#### Inclusion criteria

1. Patients with chronic tinnitus, defined as a duration of the tinnitus of 6 months or longer (2).
2. No contraindications for fNIRS scanning.
3. Individuals aged between 20 and 55 years, who are right-handed.
4. Patent external auditory canal and intact eardrum.
5. The average hearing threshold in both ears is less than 35 dB.
6. Normal expressive and comprehension abilities.

#### Exclusion criteria

1. History of drug dependence or alcohol addiction.
2. Severe central nervous system diseases or other systemic diseases such as cardiovascular and cerebrovascular conditions.
3. Organic lesions such as craniocerebral trauma.
4. Inner ear or auditory nerve diseases such as sudden deafness, Meniere’s disease, otosclerosis, or auditory neurinomas.
5. External ear or middle ear related diseases such as otitis externa, acute/chronic otitis media.
6. Patients with auditory hyperacusis.

#### Inclusion criteria for healthy subjects

1. Individuals aged between 20 and 55 years, who are right-handed.
2. All physiological indicators are within the normal range upon physical examination, with no history of functional or organic diseases, and no head injuries.
3. No family history of psychiatric or neurological genetic diseases.
4. The subject signs a paper version of the informed consent form, volunteering to participate in this trial.

#### Exclusion criteria for healthy subjects

1. Pregnant or breastfeeding women, or those who plan to conceive within the next 6 months.
2. Individuals with a history of head trauma.
3. No metallic implants in the body and no contraindications for fNIRS testing.
4. Those who are taking part in more clinical studies at the moment.

#### Allocation concealment and randomization

Suitable participants will be assigned at random to the acupuncture group or the sham acupuncture group in a proportion of 1:1 using a random stratification block design by gender and lifetime, with the Statistical Product and Service Solutions Version 25.0 (SPSS Inc., Chicago, IL, USA). An impartial administrator, uninvolved in other study protocols, will conduct this process, and the details of the list of random allocations will remain completely confidential. This randomization results will be preserved in opaque, sealed, envelopes with sequential numbers to guarantee allocation concealment.

### Blinding

The study employs a blinded evaluation method, with an uninformed third party doing the efficacy assessments regarding the group allocation. During the data summary phase, a blinded statistical analysis will be conducted, implementing the principle of separation among researchers, operators, and statisticians.

### Intervention procedures

**Figure 4:**
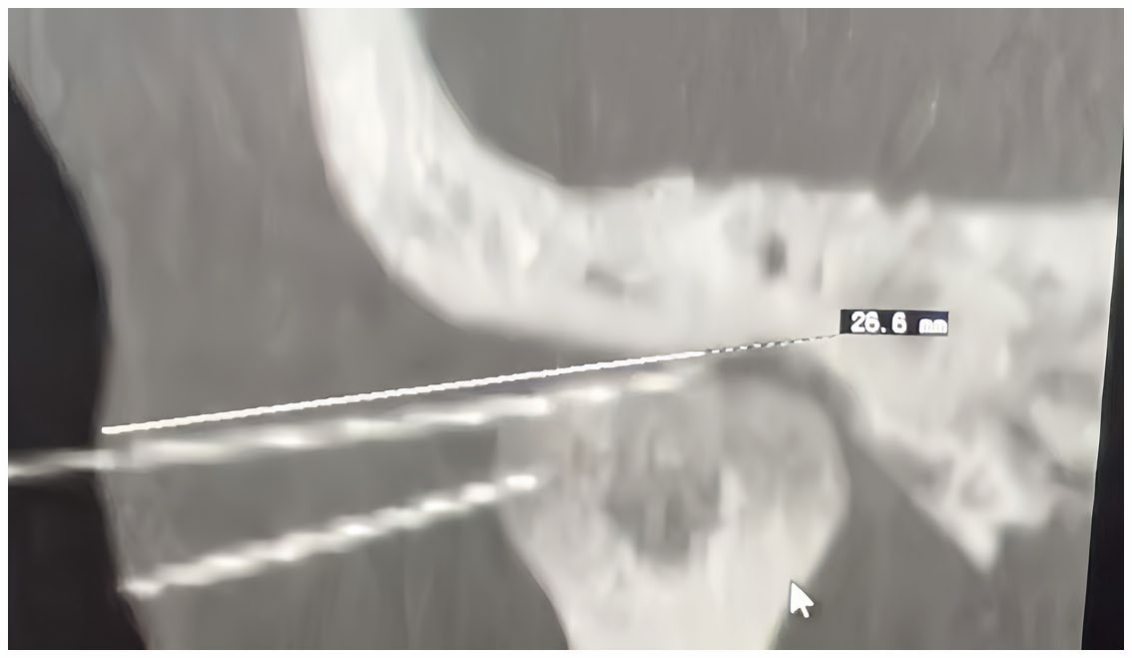
Diagram of acupuncture depth.

All participants with chronic tinnitus will receive acupuncture treatment for a total of 10 times (Acupuncture treatment is given once every 2–3 days, and it takes about 28 days for 10 treatments). We chose 10 acupuncture times based on our previous research and clinical experience of acupuncture in the treatment of tinnitus (28, 29). We believe that the methods are representative and can effectively evaluate the efficacy of acupuncture in the treatment of tinnitus. After the completion of 10 acupuncture times, we will follow up the patients within 3 months in order to more comprehensively evaluate the efficacy of acupuncture. In this context, patients in the acupuncture group should ensure the occurrence of “de qi” (a sensation of numbness, tingling, and heaviness that arises after the acupuncture needle is inserted into the body (30)) after needling the acupoints. We use CT positioning. The therapeutic group has a puncture depth of about 20–25 mm, with the needle tip reaching the temporomandibular joint (see Figure 4). Needling techniques are applied to generate “de qi” and produce a therapeutic effect. While patients in the sham acupuncture group are not required to achieve “de qi.” The acupuncture depth of the placebo group is about 5-8 mm, and the needle will not reach the treatment depth. Acupuncture techniques will not be used, and “de qi” will be not produced. What is more, in sham acupuncture, we use precise needle insertion positions and applied force to avoid interference with the actual therapeutic effect on patients. Acupuncture depth only produces therapeutic effects when it reaches a certain depth, as shown in Figure 4 (31).

### Acupuncture group

Acupuncture points prescription: The points to be used include Yifeng (affected side), Tinggong (affected side), Tinghui (affected side), Wai Guan (both sides), Zhongzhu (both sides), and Hegu (both sides) and so on. The participants in this study are licensed acupuncturists possessing more than 10 years of medical expertise of acupuncture. To guarantee strict compliance with the usual procedure for acupuncture, all professionals will undergo specific instruction on the acupuncture practices prior to the study. They will receive a booklet detailing the standardized procedures. Each acupoint will be located according to the WHO standards (32). For specific location, see Table 1.

**Table 1:** Locations of the selected acupoints.

Acupoints	Location
Yifeng (TE17)	Behind the earlobe, between the mastoid process and the mandible.
Tinggong (SI19)	In front of the tragus, when the mouth is open, at the depression.
Tinghui (GB2)	In front of the intertragic notch, at the posterior border of the condyloid process of the mandible.
Zhongzhu (TE3)	On the dorsum of the hand, between the 4th and 5th metacarpal bones, in the depression proximal to the MCP joint of the 5th digit.
Waiguan (TE5)	2 cun above the transverse crease of the wrist, between the ulna and radius bones.
Taixi (KI3)	In the depression between the medial malleolus and the Achilles tendon.
Xiawan (CV10)	2 inches above the navel, on the anterior midline.
Zhongwan (CV12)	4 inches above the navel, on the anterior midline.
Guanyuan (CV4)	3 inches below the navel, above the anterior midline.
Qihai (CV6)	1.5 inches below the navel, above the anterior midline.
Shangqu (KI17)	2 inches above the navel, with a 0.5 inch opening next to the midline.
Yindu (KI19)	4 inches above the navel, with a 0.5 inch opening next to the midline.
Hegu (LI4)	At the midpoint of the radial side of the second metacarpal bone.

Acupuncture procedure: Patients are placed in a supine position. After routine disinfection of the local skin around the ear, acupuncture needles with 40 mm in length and 0.25 mm in diameter (Hwato Brand, Suzhou Medical Instrument, China) are used for the three ear-adjacent points. Tinggong and Tinghui are punctured perpendicularly to the skin with a depth of approximately 20-25 mm. For “Yifeng point,” the needle is inserted with a slight forward tilt, meaning the direction of the puncture is toward the ear canal. The needle is inserted slowly with a depth of about 20-25 mm. The “De qi “is performed, which involves a small amplitude, low-frequency twisting manipulation with an amplitude of 180° and a frequency of less than 60 times per minute, until the patient feels a distinct sourness, numbness, and swelling in the ear that transmits into the ear canal. For the points on the limbs, acupuncture needles with 40 mm in length and 0.25 mm in diameter are used for conventional needling, and the technique of lifting, thrusting, and twirling with even tonification and reduction is applied. The Chinese Modified Massachusetts General Hospital acupuncture Sensation Scale (C-MASS) is used for quantification. All needles are retained for 30 min.

#### Sham acupuncture group

Acupuncture points prescription: In the selection of acupoint, the sham acupuncture group is the same as the acupuncture group.

Acupuncture operation: The patient is placed in a supine position, and after routine disinfection of the skin around the ears, the acupoints are punctured with 40 mm in length and 0.25 mm in diameter needle for superficial skin penetration (5–8 mm deep). The needle only needs to be inserted into the acupoints, without the need for “De qi “, and the needles are left for 30 min.

### Concurrent intervention and care

During the time frame for studying, Subjects are instructed to avoid engaging in any additional interventions which may influence the study outcomes, including, but not restricted to, medicine and behavioral treatment. If participants undergo other combined interventions for tinnitus, researchers must document pertinent facts for future examination. Nevertheless, for participants with comorbidities include hypertension, diabetes, and various other chronic conditions, they may persist with their standard medication and therapies. Researchers will document the identities of these comorbidities, drugs, and therapy in the case type of report.

### The process of fNIRS gage and record analysis

**Figure 5:**
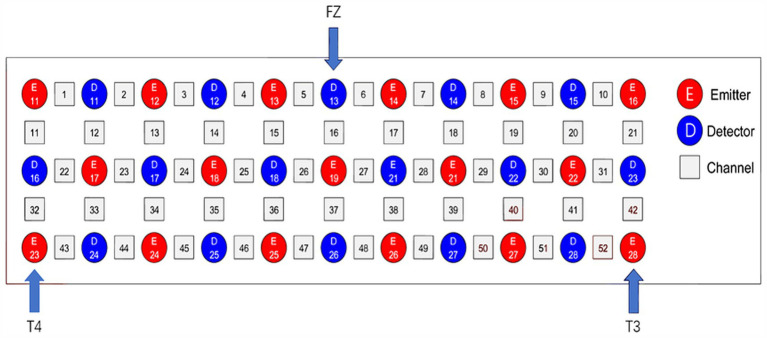
Based on the worldwide 10–20 system, Figure 2 shows the channel configuration of fNIRS. It has 52 channels (numbers), 16 detectors (blue squares), and 17 light emitters (red squares).

Our experiment’s FNIRS gage apparatus, the ETG-4000 (Hitachi Ltd., Japan), uses two wavelengths of near-infrared light (695 and 830 nm) to find differences in oxygenated hemoglobin (HbO), deoxygenated hemoglobin (HbR), and total hemoglobin (HbT). Hemoglobin variations may be monitored 2–3 cm below the surface, which corresponds to the brain’s cortex, using 3 cm between the source and the detector. The measurements will be conducted using a 52-channel fNIRS device. This system includes 33 probes (17 emitters and 16 detectors) arranged in a 3 × 11 configuration fixed to the scalp, in accordance with the international 10–20 system (33). Measurement channels are established between each emitter-detector pair, producing a total of 52 channels. See Figure 5 for the channels. The fNIRS probes will include the frontal skin and superior temporal skin. Upon stabilization of the probes, the signal power of every channel. Will be assessed. Signals from channels may be influenced by the cleanliness of the probes and obstructions, like as pile, between the probes and the scalp. Consequently, if the signal intensity of a channel is determined to be inadequate, prompt actions like scalp cleansing, probe adjustment, and hair repositioning will be implemented.

**Figure 6:**
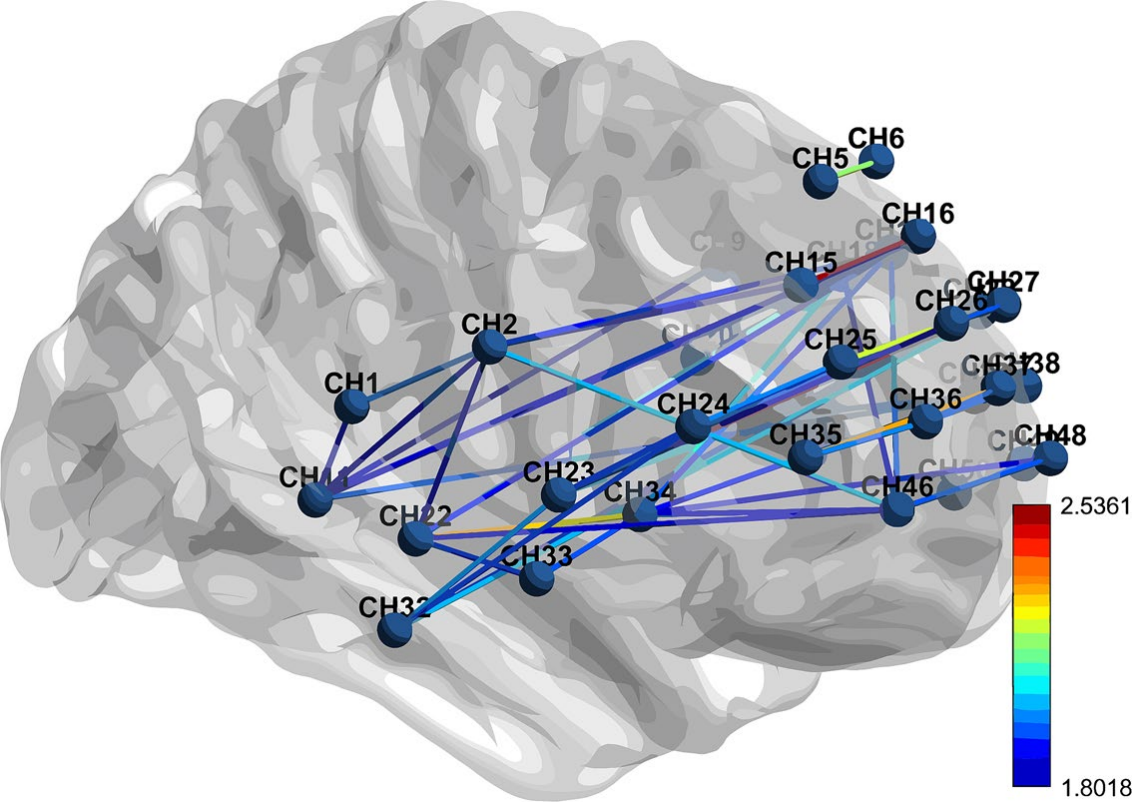
fNIRS brain functional connectivity.

Data from the ETG-4000 will be loaded into NIRS-KIT software as CSV files, a MATLAB-based software that provides researchers with an open instrument for analyzing fNIRS about data within a single suite (34). The fNIRS data will undergo preprocessing, including detrending, motion correlation, and filtering to eliminate hindrance about external variables. After preprocessing, the data will be eligible for calculating the strength of functional connectivity between regions. For specific measurement results, see Figure 6.

### ABR scanning

**Figure 7:**
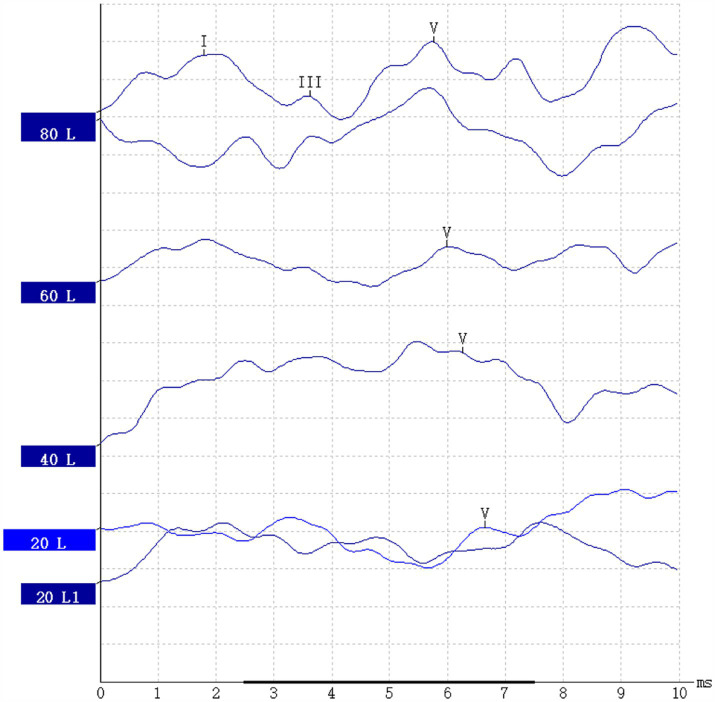
ABR report.

Auditory brainstem response (ABR) measurements will be conducted using the Danish Interacoustics Eclipse, EP25 auditory evoked potential instrument on subjects. The testing will be performed in a sound (electric) shielded chamber with an environmental noise level of ≤30 dB. The ground electrode will be placed between the eyebrows, the recording electrode on the center of the forehead, and the reference electrodes on both mastoids with a resistance of less than 5kΩ. The stimulating sound will be a click, with stimulation frequencies of 11.1 times/s and 51.1 times/s, a total of 1,024 superimpositions, and a recording time of 10 s. For specific measurement examples, see Figure 7.

### Outcome measures

Clinical Results: Clinical result metrics encompass the Tinnitus Handicap Inventory (THI), ABR testing, and fNIRS data collection. Evaluations will be carried out at baseline, after 10 treatment sessions.

THI score: The THI is a self-reported checklist that’s quick, simple. To manage and interpret, and covers a wide range of issues. It consists of 25 items, each scored from 0 to 4. An elevated THI indicates a more severe level of tinnitus.

ABR testing: The test records the peak latencies (PL), wave amplitudes, and interpeak intervals (IPL) of the subjects at various stimulation rates, as well as the differences in PL and IPL at the two stimulation rates (ΔPL and ΔIPL) to evaluate the function of the peripheral auditory nerve.

fNIRS data acquisition and processing: Cortical blood oxygen data will be collected to evaluate the central function of the subjects.

fNIRS results: fNIRS scans will be conducted at baseline, and after 10 treatment sessions. The blood oxygen concentrations measured include the concentrations of oxygenated hemoglobin (HbO), hemoglobin that has lost oxygen (HHb), and total hemoglobin (THb) in every measured channel.

fNIRS functional connectivity intensity: The intensity of functional connectivity between regions can quantify the relationships between routes or areas of interest within the fNIRS time plots. This is computed using the Pearson correlation coefficient between the average time series of any pair of channels of interest. The functional connectivity strength between channels of interest, as well as between channels of interest and other channels, is computed using NIRSKIT software. This software will help in analyzing the correlation between the time series data from different channels, providing insights into the connectivity patterns within the brain that may be associated with tinnitus and its treatment.

#### Preprocessing

Bandpass filtering is applied to the signals to remove high-frequency interference. A combination of empirical mode decomposition and wavelet transform is used to filter out baseline drift and physiological interference in the signals. A correlation-based signal improvement algorithm is employed to remove motion interference from the signals. Data is segmented according to the position of the target stimulus.

#### fNIRS signal feature extraction and modeling

Sensitive channel selection: This is done by calculating the classification accuracy rates of HbO, Hb, and THb for each channel, and accumulating these rates to amplify differences; channels are selected based on the changes in amplitude of the HbO, Hb, and tHb signals during the acquisition process.

Feature extraction and modeling from sensitive channels: Several features are extracted from the three hemodynamic signals of HbO, Hb, and tHb (the sum of HbO and Hb signals) in the sensitive channels. A support vector machine (SVM) classifier is used to establish a classification recognition model. The features that the recognition model can use mainly include mean values, fitted slopes, and approximate entropy.

### Analytical statistics

Date analysis will be done using SPSS version 26 (IBM Corporation, Armonk, NY, 2019), with a two-sided significance threshold established at 0.05. In terms of data description, continuous data is presented as mean ± standard deviation (x ± s), and categorical and ordinal data is stated as proportions (%). The intervention effects will be represented by OR and 95% CI. Data on immediate and cumulative effects of the two acupuncture groups, clinical scoring, ABR parameter analysis, and fNIRS data analysis is compared using T-tests. Permutation tests will be used, and multiple comparisons will be examined using False Discovery Rate (FDR) correction. The differences in THI scores, ABR parameters, and functional values of brain regions will be analyzed for correlation using Pearson’s correlation analysis, with inspection level *α* = 0.05.

### Sample size estimation

The formula for calculating the two independent sample sizes in Sun Zhenqiu’s “Medical Statistics” is:


$$n=1641.4\times {\left[\frac{\left(u_{a}+u_{\beta }\right)}{\sin^{-1}\sqrt{P_{1}-\sin^{-1}}\sqrt{P_{2}}}\right]}^{2}$$

In the formula, *n* represents the required sample size, the two population rates’ predicted values are, respectively, denoted by P_1_ and P_2_, while u_α_ and u*_β_* are the *u* values according to the exam level *α* and type II error probability *β*, respectively. Assuming *α* = 0.05 and *β* = 0.20, through calculation, the required sample size for each group is 30 cases. Based on a 20% exclusion rate, 36 cases should be included in each group, for a total of 72 patients.

According to the requirements for data analysis in imaging studies, a minimum of 12 healthy subjects should be included. Considering potential uncertainties such as subject head movement during scanning that may render data unusable, to ensure the reliability of data analysis, it is advisable to include 15 healthy subjects.

### Evaluation of safety

The security evaluation concentrates on the frequency of negative occurrences. Participators will be asked to report any harmful occurrences. Adverse effects associated with acupuncture mostly include soreness, hemorrhage, bruising, empyrosis, blisters, infection, and disorientation. For security evaluation, all bad events, regardless of relation with acupuncture cure, are meticulously documented and evaluated by the researchers on the adverse event form at each assessment. This will encompass the time of happening, length of symptoms, seriousness (light, moderate, or critical), management strategies, solution time, and categorization of causality (definite, probable, unlikely).

### Ethical approval and study registration

The ethics committee has approved ethics (ZSLL-KY-2024-038-01) for the trial. Before signing up, investigators will overall tell to the participants the objectives of the study, the research project, its advantages, and invisible disadvantages. Objects will hold onto the full right to think whether to join in the trial, and must sign an informed consent form before being included in the study. Throughout the research process, stringent privacy safeguards shall be maintained, and all personal and medical information will remain secret. The protocol of trial has been filed in the Clinicaltrials registry with the identification code: NCT06401993.

### Gathering and managing data

Upon acquiring completed permission forms, data will be gathered from all participants. During the experiment, participant privacy will be rigorously safeguarded. Investigators must ensure the confidentiality of the subjects. Participants will get codes as identification. Their discretion will be guarded by the researchers. Objects will be nameless, and their messages and data shall be protected in case report forms. Paper records will be authenticated and input into a password-secured electronic database. Two data entry professionals will input all information into the computer database via double data entry to guarantee data correctness. Data confidentiality is maintained, with access restricted to approved researchers alone. Participants’ research information will remain confidential and will not be revealed outside the study without their explicit agreement.

### Study monitoring and quality control

Primary cultivation will be performed prior to subject recruitment to guarantee that all researchers and pertinent personnel comply with the study protocol, hence ensuring the quality of the study. Furthermore, all result evaluators will accept standardized cultivation to consistently perform outcome evaluations and fill out case report forms. Investigators will guarantee that all acquired data is accurate, thorough, and verifiable, sourced from original files. Investigators will convene quarterly monitoring assembly to address and resolve concerns that emerge throughout the study duration.

## Discussion

So far, the neurological processes behind acupuncture in the cure of tinnitus have not been definitively established. Nonetheless, recently, a growing number of neuroimaging studies have utilized advanced technologies such as ABR and fNIRS to quest the core mechanisms about basis of therapy of acupuncture in healing tinnitus. For instance, a study based on auditory brainstem response indicated that intervention with 2 Hz electroacupuncture can enhance the recovery of ABR auditory threshold shifts (35). Additionally, a study utilizing functional magnetic resonance imaging (fMRI) has shown that acupuncture excitement can mostly alleviate the ponderance of tinnitus in subjective tinnitus patients by reducing the functional connectivity of the amygdala (36). Recently, a trial based on fNIRS found that acupuncture can increase the concentration of HbO2 in the temporal lobe of tinnitus patients and affect the motivation about auditory cortex. Furthermore, significant activation was observed in the superior temporal gyrus after acupuncture, indicating that acupuncture can regulate the motivation of the auditory area in tinnitus patients (27). Indeed, the adjustment of idiographic brain cortices might be a target for the therapeutic effects of acupuncture and the neural mechanisms underlying tinnitus treatment. Functional connectivity denotes the statistical relationships or connections between activity in various brain areas. It indicates the extent of synchronization or coherence of neuronal processes among different brain areas. Functional connectivity analysis offers significant insights into the brain’s functional architecture in response to stimuli and alterations. It boosts a comprehensive knowledge of the processes via which acupuncture enhances brain function. Recently, fNIRS has attracted increased interest by investigating the impacts of acupuncture on cerebral zones and the dynamics of inter-regional functional connectivity. The prefrontal cortex (PFC) is an area of the cerebral cortex that covers the front part of the frontal lobe. The prefrontal cortex gets information from all other cortical areas and functions to arrange and guide physical, cognitive, emotional, and social behaviors. An fNIRS study observed that, compared to the control group, the network join with bilateral PFC as a node showed importantly improved functional join during acupuncture operation (37). With its noise-free operation and strong resistance to interference, fNIRS is better to other neuroimaging skills in studying the central mechanisms of tinnitus. Therefore, we have chosen fNIRS to validate the hypotheses of this study. Concretely, we hypothesize that compared to conventional acupuncture remedy, the “Dao qi tong luo” acupuncture skill will significantly alleviate tinnitus and correspondingly observe more pronounced changes in neural activity. This will provide profound insights into the fundamental mechanics of acupuncture in the remedy of tinnitus.

Indeed, the strength of our study stem from its innovativeness. For what I can tell, there has been alone one similar exploration that utilized fNIRS to investigate the neurological processes behind acupuncture in the treatment of tinnitus (27). However, the preceding research, serving as a pilot experiment, included just 18 individuals with tinnitus and lacked a randomized controlled design. Additionally, it focused specifically on HbO2 concentration as the fNIRS-related result. Secondly, in the process of evaluating the efficacy of acupuncture and moxibustion on chronic tinnitus patients, we used ABR as one of the evaluation indicators, which makes our evaluation indicators more objective. In contrast, the objective evaluation indicators for the previous RCT study of acupuncture and moxibustion treatment of chronic tinnitus are not clear. To address these prior shortcomings, our research is structured as a randomized, assessor-blinded controlled experiment. Given the novelty of fNIRS in tinnitus and acupuncture research, our findings are partly expected to shed light on the neural mechanisms underlying the effects of acupuncture on tinnitus. Furthermore, our study aims to provide objective methods for assessing the therapeutic effects of acupuncture on tinnitus.

While our study offers several innovative aspects and methodological strengths, it is not without limitations. Firstly, as a single-center study, the sample size may lack representativeness, which could potentially impact the reliability of the results. Secondly, the severity of tinnitus among participants was not restricted in our study. This variability could influence the outcomes, and therefore, prospective research should seek more participants with similar degrees of tinnitus severity to control for this variable and reduce its potential confounding effects on the study results. Thirdly, due to technical limitations, fNIRS can only measure the hemodynamics and neural activity in the superficial areas of the cerebral cortex, and it cannot capture the activities in deeper brain regions.

## Conclusion

The results of this research are anticipated to improve our comprehension of the effectiveness and fundamental processes of acupuncture in addressing persistent tinnitus by observing the cumulative effects of the acupuncture method on patients with chronic tinnitus, it is expected to compare horizontally and vertically at multiple levels and time points, and deeply explain the mechanism of action of the acupuncture method on chronic tinnitus, providing useful insights for researchers and clinical doctors in this field.
